# Experimental and conceptual approaches to root water transport

**DOI:** 10.1007/s11104-022-05427-z

**Published:** 2022-05-19

**Authors:** Yann Boursiac, Virginia Protto, Louai Rishmawi, Christophe Maurel

**Affiliations:** IPSiM, Univ Montpellier, CNRS, INRAE, Institut Agro, 34060 Montpellier, France

**Keywords:** Root water transport, Biophysics, Modeling, Forward genetics, Pressure chamber, Root pressure probe

## Abstract

**Background:**

Root water transport, which critically contributes to the plant water status and thereby plant productivity, has been the object of extensive experimental and theoretical studies. However, root systems represent an intricate assembly of cells in complex architectures, including many tissues at distinct developmental stages. Our comprehension of where and how molecular actors integrate their function in order to provide the root with its hydraulic properties is therefore still limited.

**Scope:**

Based on current literature and prospective discussions, this review addresses how root water transport can be experimentally measured, what is known about the underlying molecular actors, and how elementary water transport processes are scaled up in numerical/mathematical models.

**Conclusions:**

The theoretical framework and experimental procedures on root water transport that are in use today have been established a few decades ago. However, recent years have seen the appearance of new techniques and models with enhanced resolution, down to a portion of root or to the tissue level. These advances pave the way for a better comprehension of the dynamics of water uptake by roots in the soil.

## Introduction

The plant root system is a highly complex and specialized organ, which serves as an anchor and forages for nutrients and water in heterogeneous soil environments. Water uptake by roots, which is the main, if not the unique, source of water for most land plants has been the object of extensive experimental and theoretical studies. Yet, this process still raises key issues for the years to come. Besides showing some apparent paradox with the laws of physics (Couvreur et al. 2021), the mechanisms which allow roots to function and acclimate under an ever-changing and challenging environment remain poorly known. Drought and flooding, in particular, are major sources of yield loss (Lynch 2007; Maurel and Nacry 2020). Thus, understanding how plants take up water may help finding solutions to mitigate the impact of these stresses. In addition, water transport in shoots has been extensively used to decipher or even predict plant responses to stresses and climate change effects (Anderegg 2015; Powell et al. 2017; Liu et al. 2020). Adding a better comprehension on root hydraulics to such knowledge will allow to expand further our possibilities of action.

During uptake by roots, water is transported radially from the soil to the root stele (Steudle 1989). Radial water flow occurs via three main pathways: apoplastic (*i.e.* water passes along the cell walls), symplastic (*i.e.* water passes from cell to cell through plasmodesmata) and/or transcellular (*i.e.* water crosses cells and cell membranes) (Maurel et al. 2015). Water is then loaded into the xylem vessels where it moves upward and is redistributed along the plant axis to the leaves and other sink organs, to support transpiration, growth and development.

In a first representation, water transport throughout a root system can be understood as the consequence of motive forces acting on hydraulic circuits. The overall force energizing the flow comes from the difference in free energy of water (aka water potential) between the xylem at the base of the root and the soil. The hydraulic properties of a root system follow from the integration, at molecular, tissue and organ levels, of all anatomical and architectural components that mediate water transport. Since the root is a usually hidden organ that is hardly accessible to the experimentation in its natural functioning mode, a lot remains to be uncovered. For example, many components of the water paths in the root have been identified qualitatively from studies at the organ level, but capturing their exact position within the whole root and their quantitative contribution still requires more efforts. The difficulties lie in the fact that a root system is an intricate assembly of molecules, structures, tissues and branches.

This review addresses the physical principles and various biological components that determine whole root water transport. After a brief theoretical introduction, we put a special emphasis on how root water transport can be experimentally measured, what is known about the underlying molecular actors, and how elementary water transport processes are addressed and scaled up in numerical/mathematical models. These approaches ultimately aim at understanding and predicting the hydraulic functioning of the whole organ. The signaling mechanisms and physiological regulations that act on root water transport have been addressed in several recent reviews (Scharwies and Dinneny 2019; Maurel and Nacry 2020; Maurel et al. 2021).

## Current experimental approaches

This section aims at providing the reader with a minimal theoretical knowledge, to understand how hydraulic properties can be experimentally determined at root cell and organ levels. A more general overview (Lambers and Oliveira 2019) or a more fundamental description of plant water relations (Dainty 1963) can be found elsewhere. The commonly used techniques are described below while the most recent methods, involving modeling, will be mentioned later on.

### Theoretical bases of root water transport

The movement of water through a semipermeable barrier can be described as:

1 $$Jv=Lp(\Delta p-\sigma RT\Delta {c}_{s})$$

where *J*v is the flow of water (m^3^.s^−1^), *L*p is the hydraulic conductance of the barrier (m^3^.s^−1^.Pa^−1^), ∆p is the hydrostatic pressure difference across the barrier (Pa), σ is the reflection coefficient, R is the gas constant (m^3^.Pa.K^−1^.mol^−1^), T is the temperature (K), and ∆c_s_ is the difference in solute concentration across the barrier (mol.m^−3^). The last terms of the equation, σRT∆C_s_, refer to the osmotic potential difference between the two compartments separated by the barrier. Since active transport of water by means of putative pump proteins plays, if any, a very marginal role (Zeuthen 2010), the movement of water is represented as passive (Tomkins et al. 2021). The equation above was first established for biological membranes (Kedem and Katchalsky 1958), and was subsequently experimentally extended to whole root systems (Dalton et al. 1975; Fiscus 1975). In the latter case, the root is assimilated to a single barrier that separates the soil solution from the lumen of the xylem vessels.

Root *L*p, also referred to as *L*o in the literature, can be described as:

2 $$Lp=L{p}_{r}.S$$

where *L*p_r_ is the root hydraulic conductivity (m.s^−1^.Pa^−1^) and S the root surface (m^2^). Other traits related to the size of the root system can be used, such as the total root length, or the root weight or volume. While measuring *L*p already provides valuable information on the hydraulic capacity of a root system, distinguishing *L*p_r_ and S allows to describe the respective contributions of the exchange surface and the intrinsic water permeability (hydraulic conductivity) of the organ.

### Whole organ

The water transport capacity of a root system can be assessed by either direct measurements or a combination of experimental measurements with inverse modeling. A quick survey of the recent literature (< 2 years) mentioning root hydraulic parameters revealed > 40 studies on species as diverse as the model plants Arabidopsis and maize, various shrubs, or the extremophile *Eutrema salsugineum*. In addition, more than half of these studies relied on measurements with pressure chambers, followed by the HPFM/HCFM (High Pressure Flow Meter/ Hydraulic Conductance Flow Meter), exudation, and finally the root pressure probe. These classical methods have been in use for many decades and shown to provide similar results (exudation was not compared though) (Li and Liu 2010). Other methods exist, such as the xylem pressure probe (Wegner and Zimmermann 2009), but are less commonly used. Here, we describe the principles and general procedure of the classical methods, and some of their limitations. A general overview can be found in Table 1.

**Table 1:**
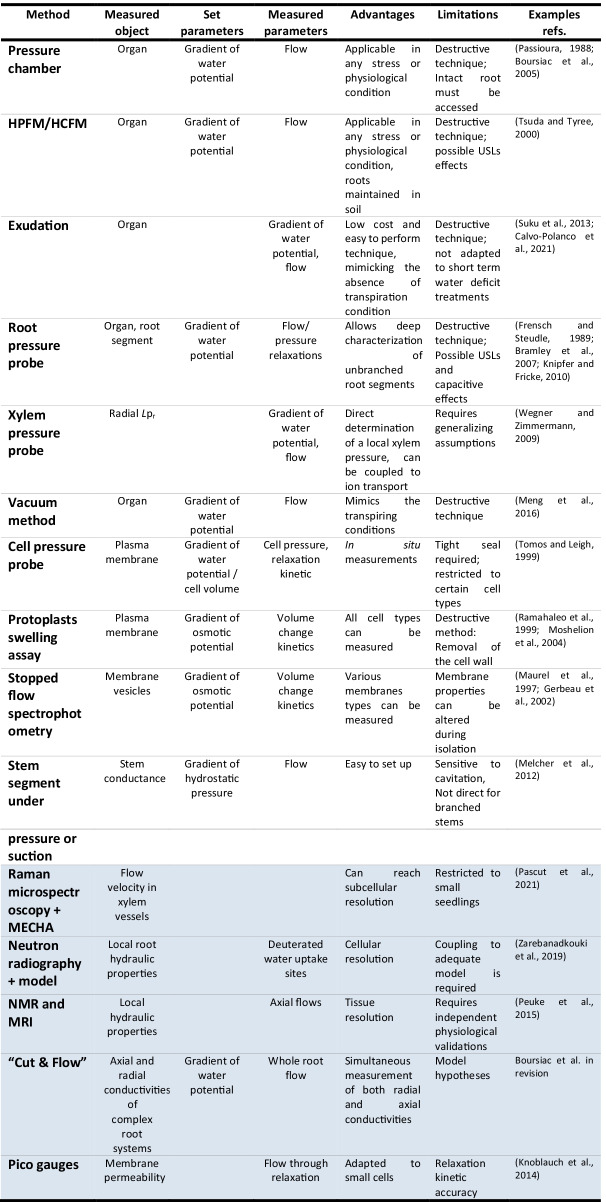
Overview of methods for measuring the hydraulic properties of roots, root segments, root cells, or membranes

Most of the methods in the first part of the table are described in section II.B; methods of the second part are described in section VI.A. USLs: Unstirred layers

The common representation behind these methods is the assimilation of a root system to a “simple barrier”, as described in Eqs. 1 and 2. Thus, *L*p_r_ represents the integration of all water transport paths present throughout the root system. They include the cell walls and other apoplastic barriers, the membranes, the plasmodesmata, the xylem vessels, and their variations along the various root branches and developmental stages. These paths cannot be deconvoluted without additional hypotheses or complementary measurements.

#### Pressure chambers

##### Description and procedure

**Fig. 1:**
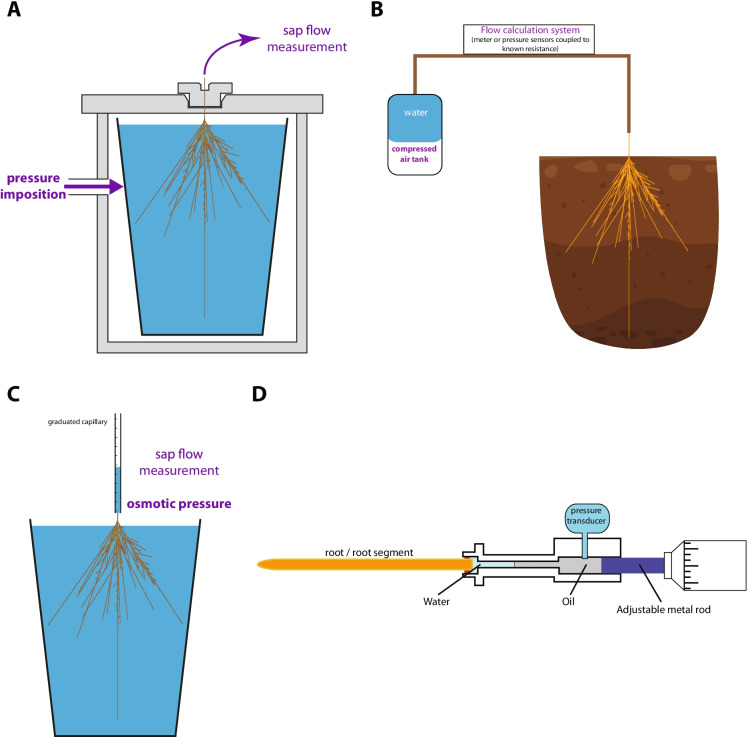
Schematic representation of experimental setups dedicated to measuring whole root water transport properties. **A**: pressure chamber, **B**: High Pressure Flow Meter (soil texture designed by brgfx / Freepik), **C**: exudation, **D**: root pressure probe ( adapted from Bramley et al. 2007). A-C: The motive force is presented bold purple, the measured flow in purple. Flow can be measured in various ways such as a graduated capillary, a balance, or a flowmeter

The technique relies on a motive force in the form of a hydrostatic pressure gradient applied to a root placed in a tank, and uses the outgoing flow to calculate *L*p (Passioura 1988). The chamber itself is designed in stainless steel and can accommodate tenth of MPa of hydrostatic pressure. The root (grown hydroponically, aeroponically, or in a pot) is placed in the tank filled with a solution. A hole in the lid allows the protrusion of the base of the root from the chamber (Fig. 1A). A critical aspect for soft tissues is to allow a tight seal around the protruding root without compressing the xylem vessels, which would otherwise generate a resistance artefact. Most of the published measurements validate a linear relationship between the gradient of water potential and the flow measured at the root base, and extract *L*p as the slope of the flow-to-pressure [*J*v(P)] relationship.

Because they rely on external imposition of a water potential gradient, the pressure chamber and related methods allow to overcome possible opposite gradients of water potential in plants cultivated in water stress conditions (drought or osmotic treatments). Therefore, it remains possible to measure a flow of sap under water deficit, and hence estimate the root hydraulic properties (Boursiac et al. 2005).

##### Limitations

General limitations of all techniques using de-topped plants are the wounding made by the cut and the interruption of communications between shoots and roots, and the disruption (at least transiently) of the water potential continuum that existed before. Root excision has been shown to trigger, within a few min, a reduction in *L*p in some plant species (Vandeleur et al. 2013). This suggests that the *L*p values obtained with these methods might underestimate the genuine conductance. It could be due for example to a cut-induced alteration in hydraulic properties due to a shift in the overall water potential with, in particular, a collapse of xylem tension. More specifically to the pressure chamber technique, linearity of the *J*v(P) relationship has to be verified in order to ensure a proper calculation of *L*p (See Eqs. 1 or 2). It is not always the case, especially at low P, when spontaneous exudation due to xylem pressure build-up within the root (Schenk et al. 2021) may deviate the *J*v(P) relationship towards higher flows. At high P, destruction of tissues due to elevated pressures may occur, or more simply the sealing might not hold. Because the root is rather impermeable to solutes, the latter may be dragged and accumulate in the vicinity of internal barriers, thereby generating osmotic counter-forces (Knipfer and Steudle 2008). It has also been argued that pressurized water may fill intercellular spaces (such as aerenchyma), hence creating new conductive paths, although this did not prove significant in soybean (Steudle and Boyer 1985).

#### Hydraulic Conductivity Flow Meter / High Pressure Flow Meter (HPFM /FCFM)

##### Description and procedure

The base of an excised root system is tightly connected to the HPFM/HCFM, which essentially consists of a circuit filled with filtered deionized water coupled to a flowmeter, and pressurized by a gas tank (air or nitrogen; Tsuda and Tyree 2000; Vandeleur et al. 2009; Ding et al. 2020; Fig. 1B). The gas allows to impose a hydrostatic pressure to the xylem, which provokes an outgoing water flow, in the reverse direction of the transpiration stream. A ramp of pressure is usually applied whilst measuring the forced flow, and artificial resistances can be added to the circuit for calibration purposes. The *L*p is calculated as the slope of the *J*v(P) relationship and can be normalized according to root size (dry weight, total length…). Alternatively, the flow measurement can be made at constant pressure, while the soil water potential is measured independently, to determine the actual water potential gradient. This technique is therefore well adapted to plants grown in soil, when the root is not easily accessible, and for a wide range of soil water potential. In particular, the ramp method is very rapid and may overcome the problems of altered *L*p_r_ upon decapitating the shoot. *L*p is however measured without normalization by the root size (eg. Jiang et al. 2020). Finally, Tsuda et al. showed a good correspondence between *L*o (plus the total plant conductance, *K*_plant_, and leaf conductance, *K*_leaf_) as measured with an HPFM and the same parameters, but measured by another technique, the evaporative method (Tsuda and Tyree 2000).

##### Limitations

The limitations of this technique are essentially the same as for the pressure chamber, due to the use of de-topped plants and the effects of pressurization on inner root water transport paths. It has also been argued that repeated pressure ramps, as well as forcing an outward water flow from the xylem, could generate unstirred layers (USLs) on the stellar side of the endodermis, which is selective to solutes. USLs effects may modify significantly the calculation of *L*p_r_ and should be taken into account. It was suggested that transient pressure steps rather than a constant pressure should be applied (Knipfer and Steudle 2008). However, it has to be noted that continuous measurements in grapevine roots did not yield flow variations by more than 10%, which were below variations observed between plants (Vandeleur et al. 2013).

#### Exudation

##### Description and procedure

Measurement of *L*o by exudation is a cost effective technique, as it just requires an osmometer. It roughly corresponds to the functioning of a root in the absence of transpiration, and hence is an adequate method to study root function in these conditions. In this configuration, a gradient of osmotic pressure is built up between the soil and the xylem vessels by the direct or indirect, but active loading of solutes (essentially mineral nutrients) into the vessels. As a consequence, soil water enters the root and the vessels, thereby pushing the sap towards the root base. The flow of exuded sap, *J*v, can be measured by various methods using a flowmeter, a graduated glass capillary, or a cotton mesh weighted over time (Javot et al. 2003; Suku et al. 2013; Fig. 1C). The water potentials of the solution and of the exuded sap have to be measured with an osmometer, in order to calculate *L*p as *J*v/∆Ѱ.

##### Limitations

A major limitation of this technique is the capacity of the excised root to maintain a significant radial osmotic pressure gradient. This requires energy for the active transport of solutes against diffusive concentration gradients. As a consequence, the flow of exuded sap can show a steady decrease, and measurements are usually not carried for more than 90 min (Suku et al. 2013). In addition, sap exudation can only be observed in the presence of an inward osmotic gradient between the soil and xylem vessels. Hence, this technique cannot be used for studying the short-term responses of plant roots to soil water deficit that would, at least temporarily, disrupt this gradient.

#### Root pressure probe

##### Description and procedure

An excised root system is connected by the stem or root base to a circuit filled with silicone oil, coupled to a pressure transducer and which volume can be adjusted by a piston. This setup allows to measure the native root pressure built in the xylem vessels by the active pumping of solutes (Steudle 1990; Knipfer and Fricke 2010; Fig. 1D). The metal rod of the device allows to impose in the xylem additional hydrostatic pressure pulses, that will be attenuated by a resulting water flow between the xylem and the bathing solution (referred to as a relaxation). It is also possible to impose “osmotic” pulses by perfusing the root with solutions of various osmotic potential. Here again, the rapid change in water potential gradient between the xylem vessels and the bathing solution provokes a water flow between the two compartments. In both cases a xylem pressure relaxation can be monitored using the root pressure probe. The kinetics of pressure adjustment is directly linked to the hydraulic conductivity of the root by the following equation:

3 $$Lp=\frac{\mathrm{ln} 2}{{T}_\frac{1}{2}\bullet {A}_{r}\bullet \varepsilon }$$

where T_1/2_ is the average half time of pressure relaxation (s), A_r_ is the total root surface area (m^2^), and ε is the elasticity of the root (root pressure variations induced by step volume changes, in Pa.m^−3^). In a variation of the method, termed “pressure clamp”, the pressure of the xylem vessels is set constant during a period (typically in the range of 2-4 min), and the flow of water pushed through the root is calculated by the displacement of the meniscus in the root pressure probe pipette. Both flow and pressure can be used to calculate the *L*p_r_, in a way very similar to that of the pressure chamber or the HPFM (Wendler and Zimmermann 1982).

The technique allows to calculate both the *L*p_r_ and the solutes reflection coefficient when imposing osmotic shocks. It is well adapted to a precise dissection of the hydraulics of an unbranched root segment (for example, Frensch and Steudle 1989).

##### Limitations

Limitations of this technique are essentially as described above for other pressure-based techniques. Furthermore, there has been a debate about the procedure that allows determining genuine *L*p_r_ values. It was shown indeed that the volume or pressure measurements during relaxation or clamps can be affected by the building of unstirred layers or by capacitive effects (Bramley et al. 2007; Knipfer et al. 2007; Knipfer and Steudle 2008). In addition, experimental errors made in determining individual parameters shown in Eq. 3 propagate upon calculation of *L*p, thereby limiting the accuracy of its measurement.

### Cell and tissue hydraulic conductivity

#### Cell pressure probe

**Fig. 2:**
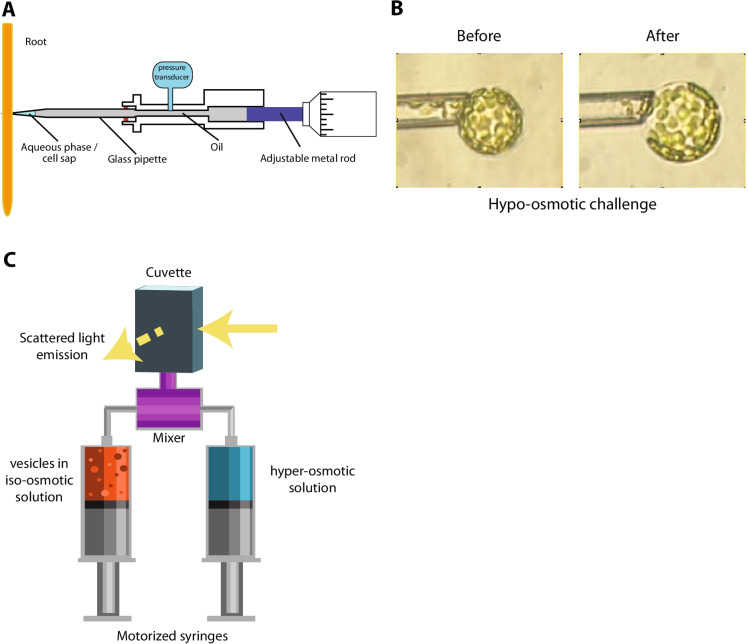
Illustrations of measurements of hydraulic properties at the cell level. **A**: drawing of a cell pressure probe, **B**: example pictures of protoplast before (left pictures) and after a hypo-osmotic shock (pipette diameter ~ 10 µm), **C**: drawing of a stopped-flow spectrophotometer

The cell pressure probe (CPP) (Hüsken et al. 1978; Steudle 1990; Tomos and Leigh 1999) consists of a tapered glass pipette, opened at the tip, filled with oil and connected to a pressure transducer and a metal rod. When a cell is impaled with the pipette, its turgor pressure compresses and repels the oil in the pipette and creates a meniscus at the cell sap-oil interphase (Fig. 2A). The CPP is thus hydraulically connected to the cell and the pressure transducer reports on its turgor pressure. Similar to the root pressure probe, the metal rod can be used to impose a series of volume changes in pulses, which trigger changes in pressure and a subsequent relaxation. The pressure relaxation kinetics (which has to be distinguished from the possible elastic relaxation of the instrument) can be recorded up to a full stabilization of the turgor through water flow between the cell and the surrounding apoplast. The hydraulic conductivity (*L*p_cell_) of a cell is given by the equation:

4 $${Lp}_{cell}=\frac{V}{A}\bullet \frac{ln(2)}{{T}_\frac{1}{2}(\varepsilon +{\pi }_{e}+{P}_{e})}$$

where V and A are the volume and the surface area of the measured cell (m), respectively, T_1/2_ is the half-time of relaxation (s), $\varepsilon$ is the elastic modulus of the cell (Pa), $\pi$_e_ is the osmotic potential of the solution (Pa), and P_e_ is the average turgor pressure of the cell (Pa).

The CPP is the only technique thus far able to experimentally measure the full hydraulic properties of a plant cell in situ. However, some cell types are not accessible to such approach due to the large size of the pipette tip with respect to cell dimension, or to the poor accessibility or visibility of the cell when deeply imbedded in inner root tissues. Clogging may also happen frequently in apoplasts rich in cell wall polymers such as waxes. Overall, impaling and pressurizing plant cells while maintaining a tight seal between the pipette and the cell remains challenging. Error propagation during *L*p_cell_ calculation can also be an issue.

#### Protoplast swelling assay

Protoplasts are usually released from roots through digestion with a mix of cell wall degrading enzymes such as cellulases and pectolyases. The isolated protoplasts can subsequently be subjected to a hypo- or hyper-osmotic shock, while the kinetics of their subsequent swelling or shrinking are monitored by videomicroscopy (Fig. 2B). In order to keep them visible throughout these processes, individual protoplasts are held in place either by a gentle suction with a smoothed glass pipette (Ramahaleo et al. 1999) or by adhesion onto a coated microscopy glass slide (Moshelion et al. 2004). The osmotic permeability coefficient of the cell, *P*_os_ (m.s^−1^), can be calculated from the initial volume change rate (dV/dt)_0_ as:

5 $${P}_{os}=\left|(\frac{dV}{dt})0\right|\frac{1}{{S}_{0}\bullet {V}_{w}\bullet \Delta {C}_{0}}$$

and the *L*p_cell_ can be calculated as:

6 $${Lp}_{cell}=\frac{{P}_{os}\bullet {V}_{w}}{R\bullet T}$$

where S_0_ is the initial surface of the protoplast (m^2^), V_w_ is the molar volume of water (18.10^–6^ m^3^.mol^−1^), $\Delta$ C_0_ is the solute concentration difference between the initial bathing solution and the hypo- or hyper-tonic solution (M), R is the gas constant (8.3144 m^3^.Pa.K^−1^.mol^−1^) and T is the temperature (K). Other methods and details about the hypotheses behind the calculations can be found in Sommer et al. (2007).

All cell types are potentially accessible to this technique, provided that protoplasts can be isolated and their origin can be unambiguously assessed. The main limitations are that protoplast manipulation is technically challenging and protoplast volume measurements have a low throughput. Most importantly, protoplast isolation (which abolishes cell turgor) and their resuspension in a hyper-osmotic solution may alter the cell membrane permeability. *L*p_cell_ measured by this method is generally lower than that measured with the CPP (Tomos and Leigh 1999; Sommer et al. 2007). This may prevent studies of regulations of water transport by osmotic signals, cell wall interactions, or by the hydrostatic pressure itself. The exact subcellular events, possibly a remobilization of intracellular membranes, that allow the swelling of protoplasts by up to 70% of their initial volume remain mysterious (Moshelion et al. 2004). A non-osmotic volume, which does not participate in water exchange but is integrated in the overall cell volume, is present in protoplasts and should be taken into account in *P*_os_ calculations (Sommer et al. 2007).

#### Stopped flow spectrophotometry

Cell membranes can be purified as small membrane vesicles from various tissue extracts, using two-phase partitioning or other techniques such as sucrose density gradient centrifugation (Gerbeau et al. 2002; Alexandersson et al. 2008). Membrane vesicles are then subjected to a hyperosmotic shock and their osmotic permeability coefficient can be deduced from the overall kinetics of volume adjustment (Horner and Pohl 2018). Due to the small size of the vesicles (< 1 µm), volume changes happen in a few dozens or hundreds of milliseconds. It is therefore necessary to use a fast kinetic technique (stopped-flow) which allows to mix, within milliseconds, minute volumes of microsomes with a hypertonic solution and monitor their subsequent behavior (Fig. 2C). The stopped-flow is usually coupled to a spectrophotometer which captures the increase in light scattering associated to the shrinkage of the microsomes (Maurel et al. 1997; Gerbeau et al. 2002). This technique has been instrumental for characterizing the water permeability of membrane fractions of different sub-cellular origins (plasma membrane, tonoplast,…) (Maurel et al. 1997) or the gating properties of plant aquaporins (AQPs; Gerbeau et al. 2002; Verdoucq et al. 2008).

#### Xylem conductance

Measurements of xylem conductance have long raised vivid debates (Melcher et al. 2012; Kim et al. 2014; Venturas et al. 2017). In particular, the occurrence of air bubbles within the vessels, which are under tension during plant transpiration, can cause dramatic changes in their conductive properties. In addition to their hydraulic conductance, xylem vessels are also characterized by their Percent Loss of Conductivity (PLC), which reports their vulnerability to cavitation. PLC is calculated from the measurement of the initial conductivity reported to the maximal conductivity of the xylem vessels. The maximal conductivity is obtained once all possible air bubbles have been expelled. The equation that allows to calculate the (maximal) xylem conductance is derived from Eq. 1, but with no osmotic flow (σRT∆C_s_) since xylem vessels are devoid of membranes (except for the immature vessels). The conductance can then be reported to the cross-sectional area of the xylem to yield the xylem specific conductivity (Melcher et al. 2012). The principle common to all experimental methods used to measure xylem conductance is to impose a given gradient of hydrostatic pressure on a stem or root section where the vessels are cut open, and measure the resulting sap flow. HPFM and equivalent devices such as Xyl’em (Bronkhorst, Netherlands, Cochard et al. 2000; Barigah and Cochard 2012) are adapted to such measurements. The root pressure probe was also used to determine the axial conductance of small, unbranched, root segments (Frensch and Steudle 1989). Since the volume of pectins in the xylem cell wall, and their impact on axial conductance, can vary significantly depending on the sap ion concentration (Zwieniecki et al. 2001), it is necessary to use a solution with a few mM of compatible salts upon injection in the vessels. However, these techniques cannot be applied to highly branched root systems, which represent complex networks of axial conductances and in which radial transport of water can no longer be negligible compared to axial water transport.

In line with the techniques presented above, a few experimental challenges remain, in order to achieve a comprehensive view of root water transport. Firstly, we miss direct measurements of the water permeability of the apoplast or other cell wall components. Although permeabilities of apoplastic tracers or particles have been reported (Carpita et al. 1979; Pecková et al. 2016), the drag of these compounds by the water flow can hardly be assimilated to the flow itself, due to their multiple putative interactions with the cell wall matrix. One avenue would be to extract native cell wall polymers (Moreira et al. 2020), and measure their permeability in vitro after thin layer or vesicle reconstitution (Kumar et al. 2007). Secondly, a comprehensive atlas of cell permeabilities is needed. While the water transport properties of a few root zones or cell layers have been characterized in detail (Peterson et al. 1993; Frensch et al. 1996; Bramley et al. 2009; Gambetta et al. 2013), we are still far from a full characterization of all cell types along the developmental axis of the root. Besides the amount of work required, another limitation is the small size of certain cell types (e.g. pericycle), which are not amenable to the CPP for example.

## Bottom up approaches: from molecules to water transport

Water availability is known to induce short-term (min to h) changes in root hydraulic conductivity (*L*p_r_) and on a longer-term (h to d) changes in root system architecture (RSA) (Maurel and Nacry 2020). In this paragraph, we look in detail at the individual elements that are involved in water transport and focus on the approaches used to demonstrate their implication.

### Aquaporins

AQPs are members of the Major Intrinsic Protein (MIP) family that facilitate the bi-directional flow of water and other small substrates across cell membranes (Chrispeels and Agre 1994; Javot and Maurel 2002; Bezerra-Neto et al. 2019). In plants, AQPs occur as multiple isoforms (at least 35, depending on species) reflecting a high diversity of cellular localizations, transport selectivity, and regulation properties. Plant AQPs are localized in the plasma membrane, endoplasmic reticulum, vacuoles, plastids and, in some species, in the membrane compartments interacting with symbiotic organisms. For a more general overview of the functions and regulations of AQPs, the reader can check various extensive reviews (Maurel et al. 2015; Laloux et al. 2018; Bezerra-Neto et al. 2019; Tyerman et al. 2021). Here we will only focus on the role of AQPs with respect to water transport. AQPs play key roles in hydraulic regulation in roots during drought stress, but also in response to stimuli as diverse as flooding, nutrient availability, temperature, or light.

The approaches used to demonstrate the involvement of AQPs in root water transport can be separated into pharmacological and genetic approaches. One of the first chemicals used for studying water transport across cell membrane was mercuric chloride (HgCl_2_). Mercurial reagents act as general AQP blockers by oxidation of accessible cysteine residue(s). This redox modification leads to the blockade and/or collapse of the aqueous pore (Daniels et al. 1996; Hirano et al. 2010). Accordingly, root treatments of hydroponically grown tomato and barley plants with HgCl_2_ resulted in a marked reduction of *L*p_r_ measured with pressure chambers, which was tentatively reversed using a reducing treatment with dithiothreitol (DTT) (Maggio and Joly 1995; Knipfer et al. 2011). Another chemical used to block AQP activity is sodium azide (NaN_3_). NaN_3_ treatment of Arabidopsis roots induced a marked inhibition of pressure-induced water flow (87%) (Tournaire-Roux et al. 2003). One of the most established modes of action of NaN_3_ is to bind to the metal cofactors (i.e. heme, a_3_ and Cu_B_) of cytochrome *c* oxidase (complex IV) (Yoshikawa et al. 1998; Fei et al. 2000). This results in a strong impairment of cell respiration, generating a cytosolic acidification (Zhang and Tyerman 1991; Kamaluddin and Zwiazek 2001). This acidification triggers the protonation of a histidine residue that is highly conserved in the second intracellular loop of plasma membrane AQPs (PIPs); this protonation in turn provokes the closure of the AQP pore (Tournaire-Roux et al. 2003). A similar inhibition can be obtained by cell acid loading using permeable weak acids such as propionic acid. Interestingly, cytosol acidosis was identified as the primary cause of *L*p_r_ inhibition in anoxic conditions (Tournaire-Roux et al. 2003). Divalent-cations are also important for regulation of AQPs (Zhang et al. 2013). As shown by measurements using a CPP, Arabidopsis suspension cells treated with Ca^2+^ lowered their *L*p_cell_ as a result of plasma membrane water channel inhibition (Gerbeau et al. 2002). In contrast to protons, divalent cations act through binding to residues located on the N-terminal tail and first intracellular loop of PIPs (Gerbeau et al. 2002; Tournaire-Roux et al. 2003; Verdoucq et al. 2008).

Besides information on the gating properties of plant AQPs, these inhibiting treatments can be used to probe AQP function in whole roots. For instance, a combined usage of different inhibitors (mercury, propionic acid and azide) in 13 Arabidopsis natural accessions allowed to show that the AQP contribution to *L*p_r_ varied from 30 to 77%, according to the genetic background (Sutka et al. 2011). Root water transport is also known to be responsive to nutrient availability. For example, wheat (*Triticum aestivum* L. cv. Chinese Spring) plants grown under nitrogen or phosphorus deprivation (for 5 and 7 d, respectively) showed an inhibition in root *L*p that was similar to that observed after a HgCl_2_ treatment. After nutrient resupply, *L*p recovered to values similar to those of control plants. This work gave the first functional proof of AQP implication in water uptake during plant response to nutrient availability (Carvajal et al. 1996).

In addition to pharmacological approaches, several genetic approaches, relying on gain or loss-of-function, have revealed the contribution of AQPs to *L*p_r_. Insertion mutagenesis has been used to generate AQP knockout mutants in several plant (Javot et al. 2003; Ding et al. 2020)*.* For instance, Arabidopsis *AtPIP2;2* is predominantly expressed in roots, with strong expression in the cortex, endodermis, and stele of elongated root segments (Javot et al. 2003)*.* Its genetic disruption using *Agrobacterium* transferred DNA (T-DNA) revealed that *AtPIP2;2* contributes by 25–30% to the *L*p_cell_ of the cortex. A 14% decrease in *L*p_r_ with respect to wild type was observed with the exudation method whereas not difference was measured with pressure chambers (Javot et al. 2003)*.* The same genetic approach was used to disrupt *AtPIP1;2* showing that the corresponding isoform significantly contributes to the hydraulic conductivity of both roots and rosette, therefore representing a key component of whole-plant hydraulics (Postaire et al. 2010). Yet, and at variance to *pip2;2* mutants, *pip1;2* mutants showed a reduction in *L*p_r_ when measured with pressure chambers but not using osmotic methods. Thus, identifying the functional role of aquaporins may be a complex task. In maize (*Zea mays*), insertion of a Mu transposon was used as another approach to knockout a single gene (*ZmPIP2;5*) encoding here the most highly expressed root AQP. Mutant plants for *ZmPIP2;5* showed a decrease in cortical *L*p_cell_ and *L*p_r_ by 63% and ~ 60%, respectively (Ding et al. 2020).

The antisense technique was also used for targeted disruption of AQP mRNAs in Arabidopsis and tobacco (Kaldenhoff et al. 1998; Siefritz et al. 2002). In the former species, the antisense lines showed a shoot development similar to control plants, but the root system proved to be five times more developed than in wild type plants. Xylem pressure measurement suggested that the increase of root mass in antisense plants compensates for the reduced water permeability of root cells in order to ensure a sufficient water supply to the aerial part (Kaldenhoff et al. 1998). Another example comes from the study of *NtAQP1* from *Nicotiana tabacum*, a gene expressed in all organs but with the highest levels in the root. Comparison of antisense with control plants provided evidence for the importance of AQP-mediated water transport in whole-plant water relations. As a consequence of *NtAQP1* silencing, droughted plants were impaired in their ability to restore leaf turgor upon rewatering, suggesting a defect in root water uptake and/or redistribution in leaves (Siefritz et al. 2002).

Besides artificial microRNA (amiRNA), which however was not used for root AQPs (Sade et al. 2014), virus-induced gene silencing (VIGS) is another reverse genetics technology that can produce a rapid, sequence specific, knockdown phenotype for a target gene (Burch-Smith et al. 2004). This technique was used to study the *Ps*PIP2;1 isoform of pea (*Pisum sativum*). Root and leaf hydraulic conductivities were significantly reduced in *PsPIP2;1*-silenced plants compared with control plants (Song et al. 2008).

Finally, over-expression of AQPs has also been used to address their role in plant-water relations. The results obtained with this technique are complicated to interpret, because changes in plant architecture may occur simultaneously to changes in tissue hydraulics. A typical example is given by the overexpression of *Hv*PIP2;1 in barley, which resulted in an increase in *L*p_r_ by 40%, together with an increase in shoot-to-root mass ratio by 50% (Katsuhara et al. 2003).

### Plasmodesmata

Although plasmodesmata have been proposed to be responsible for symplastic transport of water, there is to date no study that has directly addressed their role in root water uptake. While examining the effects of O_2_ availability and NaN_3_ on cortical cells of wheat roots, Zhang and Tyerman (1991) observed a reduction in apparent cell osmotic volume that was attributed to a decrease in hydraulic coupling of cells, likely due to a plasmodesmata closure. The decrease in cell *L*p that was observed in parallel was also interpreted as a sign of plasmodesmata closure. Yet, we now know that the latter effects are likely due to pH-dependent gating of AQPs (Tournaire-Roux et al. 2003) and that this physiological context is likely to increase the size exclusion limit of plasmodesmata (Cleland et al. 1994).

### Apoplastic root barriers

It is now established that cell membranes represent highly selective barriers for water *vs.* solute transport, while the apoplast would be much less selective. Yet, the respective contribution of these two pathways during root water uptake remains under debate. In particular, vascular plants display two apoplastic barriers that prevent uncontrolled passage of water and nutrients through the roots, whilst also acting in plant protection against stresses. These barriers are located in the apoplasm of the endodermis and exodermis where they appear as lignin-made Casparian strips (CS), suberin lamellae and thickened walls (Hose et al. 2000; Enstone et al. 2002; Barberon et al. 2016). The endodermis, which corresponds to the inner cell layer of the cortex surrounds the central vasculature and plays a key role in plant nutrition due to its capacity to form a selective barrier for water and nutrients (Barberon 2017). The exodermis, which is located underneath the epidermis and can be considered as one type of hypodermis harbors CS in its anticlinal cell walls (Peterson and Perumalla 1990).

Several approaches have demonstrated how these structures have specific roles in the radial transport of water and solutes. For instance, the manual injury of a suberin-deprived endodermis in hydroponically grown maize roots showed that the endodermis with CS represents a barrier for solutes but not for water (measured with the root pressure probe) (Peterson et al. 1993; Steudle et al. 1993). From the quantification of several permeability parameters along the root, it was concluded that suberin represents a major barrier for water flow (Frensch et al. 1996). Genetic approaches using mutants with altered suberin content or defective CS also confirmed that suberin is a direct barrier to water transport while CS is rather involved in preventing passive solute diffusion through the root (Ranathunge and Schreiber 2011; Calvo-Polanco et al. 2021). However, CS integrity is tightly surveilled by the CIF/ SGN3 signaling pathway (Doblas et al. 2017; Nakayama et al. 2017) and any alteration in CS permeability can result in the deactivation of AQPs, which will indirectly result in a reduction in *L*p_r_ (Wang et al. 2019).

### Xylem

Historically, vessels have been considered as thin cylindrical tubes that are interconnected through perforated cross walls. The Hagen-Poiseuille’s law indicates that the quantity of water flowing through such tubes is proportional to the fourth power of the tube radius (Lewis and Boose 1995; Zwieniecki et al. 2001). However, both the internal anatomy and the interconnections between xylem ducts are variable. In particular, the ion concentration of the sap impacts the swelling of hydrogels such as pectins, which may alter the vessels diameter as well as affect the permeability of the pit membranes (Zwieniecki et al. 2001; Zwieniecki 2003). Therefore, the xylem is more complex than a series of parallel straight-walled tubes, and its hydraulic conductance may have been overestimated. Only a few studies have experimentally measured xylem conductance in roots. Among these, the axial hydraulic conductivity of root segments of maize was directly measured in excised root segments or was estimated from cross-sections using Hagen-Poiseuille's law. The authors observed a discrepancy by a factor of 2 to 5 between the two approaches, the later providing the higher estimates (Frensch and Steudle 1989). An even greater factor was found in prunus (Vercambre et al. 2002).

Since multiple radial and axial pathways act in concert to determine whole root hydraulics, the respective contributions of these pathways, rather than their absolute conductance, also have to be considered. In particular, the relative limitations of radial *vs.* axial transport affect the hydraulic architecture of the root and determine its major sites for water uptake (Zwieniecki et al. 2002). It has been argued that xylem vessel conductance is orders of magnitude greater than radial conductance in maize, barley or lupin (Steudle and Peterson 1998; Bramley et al. 2009). This would point towards a major limitation of water uptake by the radial pathway. Such idea may not hold in Arabidopsis where alterations in xylem differentiation was associated to a drop in the root hydraulic conductance (Lefebvre et al. 2011; Tang et al. 2018; Boursiac et al. in revision).

In conclusion, the present section shows that many cellular structures and molecular components involved in root water transport have been validated, though at different resolution. In particular, the exact role of plasmodesmata remains to be confirmed. In addition, most of functional validations were performed at the whole root level. Thus, future research will have to map and quantify the effect of the actors at tissular and cellular levels. This remark echoes our conclusion on experimental methods where better resolutions at tissular and cellular levels is also needed.

## Models

**Fig. 3:**
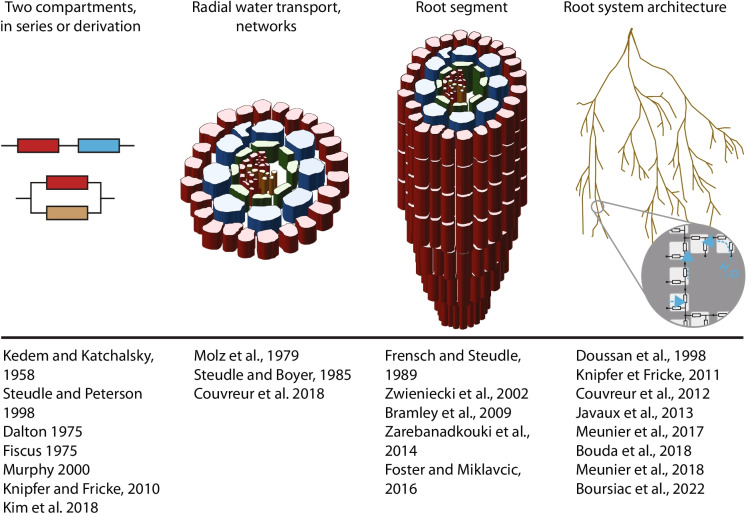
Representative root water transport models in relation to their structure or scale. The first column essentially refers to models that use a two compartments analogy (soil and xylem), representing either the whole root system or radial water transport. The second column presents models as a network of resistances that allow to recapitulate radial water transport. The third column (“root segment”) cites models that address the hydraulic function of a root segment or of an unbranched root (it may share similarities with the first column). Finally, the last column presents models that aim at calculating the water flow over an entire and highly branched root system. Note that this figure presents a non-exhaustive list of models

“To fully understand root system hydraulics […], comprehensive studies at different scales are required (cells, organs, and whole roots)” (Bramley et al. 2009). Mathematical modeling of water transport has been key to this understanding by providing a theoretical framework for the interpretation of experimental measurements as well as for bridging scales. We note that specific formal representations and mathematical models of water transport have been developed at each of these scales (Fig. 3): water and solute transport across membranes based on the thermodynamics of irreversible processes (Kedem and Katchalsky 1958; see above), radial water transport through the interplay of apoplastic/cell-to-cell pathways with, in particular, the composite transport model (Steudle and Boyer 1985; Steudle and Peterson 1998; Couvreur et al. 2018; Kim et al. 2018), and integrated water transport in root segments (Frensch and Steudle 1989; Zwieniecki et al. 2002; Bramley et al. 2009; Zarebanadkouki et al. 2014; Foster and Miklavcic 2016) or whole root architectures using equivalent hydraulic network representations (Doussan et al. 1998a; Knipfer et al. 2011; Couvreur et al. 2012; Javaux et al. 2013; Meunier et al. 2017, 2018; Bouda et al. 2018). A current challenge is to couple these different models and integrate degenerate representations of lowest scales in the most integrative models.

Concerning the whole root scale, one of the foundation was the assimilation of water transport to a catenary process (a network of resistances in series or in parallel) and the analogy to the Ohm’s law, where the water transport is directly proportional to a potential difference (Honert 1948). This view was developed further by Doussan and his colleagues in 1998 (Doussan et al. 1998a, 1998b). In these works, the root was “split” into small elemental units that are defined by their axial and radial water transport capacities. These so-called root segments were assembled into a “hydraulic tree” in order to reconstitute the full root system architecture. This type of model, referred to as a Functional Structural Plant Model (FSPM), bridges scales in that it reconstitutes the whole root transport properties from units that are just a few cells long. Thus, this approach allows to obtain the water transport capacity of the whole root, while resolving the gradient of water potential within the RSA and the “elementary” flow uptakes.

In their diversity, root models are designed to answer specific questions, the non-relevant aspects being simplified to reduce the model complexity. For example, some models have focused on the hydraulics of single root segments (Frensch and Steudle 1989; Zwieniecki et al. 2002; Zarebanadkouki et al. 2014; Foster and Miklavcic 2016) or used a simplified representation of the hydraulics of the root system (Knipfer et al. 2011). Analytical solutions of water flow equation have also been used to create a model of whole root hydraulic architecture (Meunier et al. 2017), where the lateral roots are encoded by their respective *L*p and inter-branching distance. Despite these approaches, the respective limitations of axial and radial conductance and their precise contribution to determining the preferential sites for water uptake still remain uncertain. Models of water uptake in entire 3D root networks have recently been developed to address these questions (Bouda et al. 2018).

More than simply recapitulating the known hydraulic parameters of a root system, models are also extremely useful for testing the quantitative impact of these parameters on root water uptake. For instance, Meunier et al. (Meunier et al. 2017) or Boursiac et al. (in revision) observed that a homogeneous increase in xylem conductance changes the sensitivity of the root system to variations in radial hydraulic conductance, indicating that these two conductance components are somewhat co-limiting.

This survey indicates that many architectural and hydraulic parameters can now be integrated into and tested through current root models. Yet, we insist that as many input parameters as possible must be experimentally measured for models to match with reality. This realization brings us back to the technical challenges mentioned in the previous sections.

## Top down approaches: from water transport to molecules

Besides reflecting specific candidate genes or cellular functions for water transport, as seen above, the hydraulic properties of plant roots (*L*p and *L*p_r_) can also be regarded as plant traits. As such, forward genetic approaches can be applied to find molecular players involved in regulating root water transport. Essentially two strategies have been used to identify Quantitative Trait Loci (QTL) responsible for natural variation of *L*p and *L*p_r_: the bi-parental QTL linkage mapping, and the Genome Wide Association (GWA) mapping. The bi-parental approach depends on the genetic recombination and segregation events in the progenies of the two parents during the construction of the mapping population. Therefore, a major limitation in the bi-parental QTL mapping is that only the allelic diversity within the parents can be exploited, which consequently affects the genetic mapping power. Over the past decade and as a result of improvement in genomic sequencing, GWA has become a method of choice to explore at whole the intraspecific genetic variation present in hundreds of lines. GWA studies make use of the variations among the sequenced genomes and correlate them with the phenotypes of the sequenced accessions to identify causal polymorphisms. They have therefore allowed to overcome the limited number of alleles addressed in bi-parental QTL linkage mapping and proved to be a prevailing approach in identifying QTLs of complex traits (Atwell et al. 2010). However, due to the population structure and genetic relationships, GWA studies are prone to false positive associations (Bergelson and Roux 2010). To overcome the limitations in both bi-parental and GWA mapping approaches and to achieve the most accurate QTL results, some studies combined the two analysis methods and have successfully mapped loci to associated traits in maize, rice and Arabidopsis (Lou et al. 2015; Rishmawi et al. 2017; Wang et al. 2018).

The aforementioned QTL strategies require a massive phenotypical analysis. Since the analysis of root hydraulics in a huge number of plants is laborious and requires specialized techniques, very few studies tackled the genetic analysis of *L*p or *L*p_r_ using QTL mapping. The distinct root hydraulic profiles among 13 Arabidopsis accessions (Sutka et al. 2011) and variable water uptake ability among 20 rice accessions (Gowda et al. 2012) steered further QTL based analysis to identify the main genetic component regulating root hydraulics and water uptake. QTL analysis of the Arabidopsis bi-parental Col-0 X Bur-0 population has prompted to the identification of three *L*p_r_ QTLs (Shahzad et al. 2016). Further fine mapping of one of those QTLs has led to the molecular cloning of Hydraulic Conductivity of Root 1 (HCR1), which encodes a raf-like MAP3K protein kinase (Shahzad et al. 2016). Analysis of *hcr1* mutants showed that these mutants exhibit an increase of 15% in *L*p_r_ compared to the wild type plants indicating that HCR1 negatively controls *L*p_r_. Noticeably, this phenotype was only observed under combined hypoxia and potassium sufficient conditions, with maximal HCR1 mRNA and protein accumulations obtained under these conditions. Additional transcriptomic analysis performed on *hcr1* mutants revealed that HCR1 acts in coordinating the core anaerobic transcriptional response with potassium availability, one of its downstream effect being root hydraulic regulation. It was proposed that HCR1 activation in the presence of potassium promotes plant acclimation to flooding stress, thereby providing an enhanced growth capacity during the recovery phase (Maurel and Nacry 2020).

In a relatively more comprehensive approach, GWA study performed on 143 Arabidopsis accessions identified two QTLs that were associated with *L*p_r_ variation (Tang et al. 2018). Detailed analysis of the candidate genes surrounding one of associated SNPs successfully revealed XYLEM NAC DOMAIN 1 (XND1) as a regulator of *L*p_r_. The *xnd1* mutants exhibited higher *L*p_r_ values compared to the wild type indicating that XND1 acts as a negative regulator of root hydraulics. Detailed analysis showed that XND1 is a NAC transcription factor that is preferentially expressed in the xylem and negatively regulates xylem differentiation (Tang et al. 2018).

Even though there have been recent achievements in molecular cloning of *L*p_r_ regulators in Arabidopsis (Shahzad et al. 2016; Tang et al. 2018), little work has been done thus far to resolve the genetic basis of root hydraulics in crop plants. In rice, the strategy of performing QTL mapping on recombinant inbred lines (RILs) derived from two cultivars was implemented and QTLs for exudation rate and root hydraulics were detected (Adachi et al. 2010; Yamamoto et al. 2016). However, those studies lacked an in depth analysis and molecular characterization of single genes for the obtained QTLs.

## Latest developments and perspectives

### Latest experimental developments

While most of the current studies on root water transport make use of well-established techniques that have been around for several decades, we note recent efforts to develop new techniques for measuring water transport at smaller scales. In addition, these new techniques rely on the coupling of exquisite experimental measures with inverse modelling.

For instance, the use of Raman microspectroscopy detection of deuterated water after pulse/chase experiments, coupled to an extension of the MECHA model for radial water transport (Couvreur et al. 2018), allowed to calculate the flow velocity in xylem vessels of Arabidopsis seedlings (Pascut et al. 2021). In other approaches, neutron radiography visualization of deuterated water was used to image water uptake sites, which were fed into model-assisted computation approach. These combined analyses were used to infer the corresponding local hydraulic properties and water potential (Zarebanadkouki et al. 2016, 2019).

NMR and Magnetic Resonance Imaging (MRI) approaches also provide interesting perspectives to measure in vivo transport pathways, and membrane and tissue permeabilities. Whereas axial volume flows along vascular tissues have been nicely monitored using MRI (Peuke et al. 2015), the effective diffusion of water within tissues as estimated by NMR (Velikanov et al. 2015) is indirectly related to water permeability but does not reach yet the cellular resolution expected in such studies.

Boursiac et al. (in revision) recently elaborated an updated version of the pressure chamber technique that consists in measuring water flow through a mature root system in a pressure chamber upon various cuts at the base or at the tip. Adapted from single branch experiments (Zwieniecki et al. 2002) to highly branched root systems, this “cut and flow” method, coupled to a functional modeling of the root, allowed to calculate both axial and radial properties in a single experiment.

At the cell level, Knoblauch et al. ( 2014) developed an alternative to the CPP, especially suited to low volume cells. These pico gauges are made of clogged glass pipettes filled with minute volumes of oil at the tip. Measurement of the meniscus displacement and recovery upon impaling a cell, together with the knowledge of the pipette volume and the oil compressibility, should allow to calculate the cell membrane permeability.

Finally, Heymans and collaborators proposed an almost purely computational approach to determine local hydraulic properties, whereby a first model (GRANAR) is used to reconstitute the anatomical structure of a cross section of root, that is then imported into a radial hydraulic model (MECHA). The later computes local tissue conductances from literature-based conductivities values of the apoplastic, symplastic, and transcellular paths of water (Heymans et al. 2021).

### Without a priori approaches: pharmacological screening and chemical genetics

Forward genetics approaches can reach their limits when dealing with, for instance, genes with redundant functions or the mutation of which is lethal (Serrano et al. 2015). Chemical genetics can provide a means to circumvent these limitations. In this approach, the effects of thousands of molecules with well-characterized structures are randomly tested on a trait of interest. This approach allowed the successful identification of compounds capable of inducing root growth in Arabidopsis and rice (Dickinson et al. 2019), regulating cell expansion (Park et al. 2009), or enhancing resistance against pathogenic *Pseudomonas* bacteria in Arabidopsis (Noutoshi et al. 2012). Additionally, other studies have uncovered compounds that can be used as tools for dissecting auxin biosynthesis and transport, thereby improving our understanding of root development (Nishimura et al. 2012; Zhu et al. 2019).

Even though plenty of chemical compounds are applied as pesticides or herbicides on several plant cultures, the effects of these or related molecules on root hydraulics has never been reported, to the best of our knowledge. More generally, the identification of chemicals specifically acting on root water transport, and the dissection of their mode of action using physiology or genetics, could provide new avenue to understand and manipulate this important function.

### Root water transport and crop improvement

Some recent studies have explored the strategy of increasing crop productivity under different environmental conditions by maximizing the root/rhizosphere efficiency (Shen et al. 2013). On one hand, some scientists believe that a deep and thick primary root adequately complemented by either laterals or seminal roots would enhance the acquisition by crops of nutrients and water that are available in deep soil (Lynch 2013). Multiple QTLs associated with root growth under drought stress have thereby been identified in rice, maize and other crops (Kim et al. 2020; Siddiqui et al. 2021). On the other hand, the efficiency of having bigger roots to absorb more nutrients and water has been questioned. A bigger root system requires a strong carbon investment in the soil, which would reduce the plant’s capacity to fix carbon in the harvested aerial organs, thereby reducing yield (Garnett et al. 2009). This cost of bigger root is evident in QTL studies where the relationship between root size and yield at low N is not clear and might be negative (Gallais and Coque 2005). An alternative strategy of enhancing water uptake without affecting root growth, through increasing *L*p_r_, could provide a solution for the trade-off between plant growth and water acquisition. However, this may not be achieved without caveats such as increased xylem vessels diameter which would increase the sensitivity to cavitation, or enhanced water loss under low soil water potential (Brodribb et al. 2015).

Even though it is generally believed that improving root hydraulics can enhance the plant growth capacity, its impact on plant responses to environmental stresses is far from fully established. Indeed, plant responses to drought may depend on species and climatic scenarios (Maurel and Nacry 2020). For example, studies have shown that species sensitivity to drought stress is essential in shaping the species pattern distribution in tropical forests (Engelbrecht et al. 2007; Choat et al. 2012). Moreover, the plant hydraulics was used as a parameter to understand and predict the dynamics of tropical vegetation under different water stresses (Oliveira et al. 2021). Therefore, it is necessary to pursue identifying the physiological and genetic regulators of *L*p_r_, in both model and crop plants, and under various environmental conditions. This will surely assist breeding and nature preservation programs to face the challenges of climate change.

### Conclusive remark

Root water transport studies have been based on a set of robust physiological methods and theories, most of which are a few decades old. From the early 90’s, the expansion of molecular biology in plant water transport research has brought a lot of components to the list of potential actors. Yet, many of these still require to be properly assessed through experimentation. A major limitation is the biological and topological complexity of plant root systems, which combine many tissues at various developmental stages in a branched system. The last decade has seen promising new avenues with the increasing prevalence of modeling, forward genetic approaches, and new model-aided experimental techniques. All these developments will hopefully reach their full potential in the coming years. Altogether, these advances draw a picture in which experimental and conceptual approaches on root water transport are ready to embrace the complexity and dynamics of water uptake in natural soil conditions.
